# UNDERSTANDING CHALLENGES AND STRENGTHS EXPERIENCED BY CHINESE AMERICAN FAMILY CAREGIVERS OF PERSONS WITH DEMENTIA

**DOI:** 10.1093/geroni/igac059.2346

**Published:** 2022-12-20

**Authors:** Jessica Cassidy, Kathy Lee

**Affiliations:** University of Texas at Arlington, Arlington, Texas, United States; University of Texas at Arlington, Coppell, Texas, United States

## Abstract

Chinese American family caregivers of persons with dementia are often challenged by barriers to utilize caregiving support services due to language barriers, minimal understanding of dementia, limited numbers of culturally competent health care providers, as well as conflicting cultural norms, such as the disease related stigma. Subsequently, a delayed diagnosis and a hesitance to seek social support services and formal resources are prevalent among Chinese American families caring for persons with dementia. However, the resilience of these caregivers is often noted as a strength of this sub-population. We aimed to identify the challenges and strengths expressed by Chinese American family caregivers caring for persons with dementia by exploring their caregiving experiences. Using a qualitative study design, we conducted semi-structured, in-depth interviews with 26 Chinese American family caregivers of persons with dementia during the midst of COVID-19 pandemic. The data were analyzed using thematic analysis. Two themes emerged as challenges: lack of knowledge regarding early signs of dementia and family caregivers’ social isolation and loneliness. Two additional themes emerged as demonstrated strengths: utilizing technology throughout the caregiving trajectory and family caregivers’ helping behaviors. Our findings indicate potential to promote disease-related education and reduce feelings of social isolation and loneliness among Chinese family caregivers caring for persons with dementia by using technology and peer-led approaches. The examined coping strategies of Chinese American caregivers may be leveraged across disciplines to increase disease related knowledge, rates of early diagnosis, as well as the utilization of formal supports and resources.

